# Insights into the Topology and the Formation of a Genuine ppσ Bond: Experimental and Computed Electron Densities in Monoanionic Trichlorine [Cl_3_]^−^

**DOI:** 10.1002/anie.202013727

**Published:** 2020-12-01

**Authors:** Helena Keil, Karsten Sonnenberg, Carsten Müller, Regine Herbst‐Irmer, Helmut Beckers, Sebastian Riedel, Dietmar Stalke

**Affiliations:** ^1^ Georg-August-Universität Göttingen Institut für Anorganische Chemie Tammannstrasse 4 37077 Göttingen Germany; ^2^ Fachbereich Biologie, Chemie, Pharmazie Institut für Chemie und Biochemie—Anorganische Chemie Fabeckstrasse 34–36 14195 Berlin Germany

**Keywords:** charge density distribution, charge shift bonding, computational chemistry, halogen bonding, trihalogen anion

## Abstract

So far, several publications have discussed the bonding concepts in polyhalides on a theoretical basis. In particular, the trichlorine monoanion is of great interest because its structure should be symmetrical and show two equidistant Cl−Cl bonds. However, apart from matrix‐isolation studies, only asymmetric trichlorine anions have been reported so far. Herein, the trichlorine monoanions in 2‐chloroethyltrimethylammonium trichloride [NMe_3_EtCl][Cl_3_], **1**, tetramethylammonium trichloride [NMe_4_][Cl_3_], **2**, and tetrapropylammonium trichloride [NnPr_4_][Cl_3_], **3**, are analysed. High‐resolution X‐ray structures and experimental charge density analyses supported by periodic quantum‐chemical calculations provide insight into the influence of the crystalline environment on the structure of these [Cl_3_]^−^ anions as well as into the progress of the bond formation between a dichlorine molecule and a Cl^−^ anion.

## Graphical abstract

For the first time, the 3c–4e bond of a symmetric trichlorine anion [Cl]^−^ was characterized by experimental charge density investigations and compared to two asymmetric cases. Quantum‐chemical DFT calculations for both periodic crystals and isolated molecules/ions flank those studies. There appears to be a smooth transition from the asymmetric to the symmetric compound.

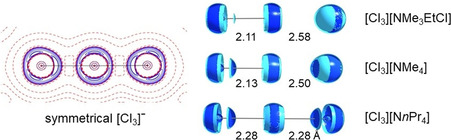

The concepts of chemical bonding, bond formation, and cleavage are of central importance in chemistry and still subject to ambitious and challenging experimental and theoretical research. The traditional view in terms of a covalent, ionic, and metallic bonding^[1]^ has been questioned, partly because the distinction between these bond types is not always clear‐cut, and in some simple diatomic molecules of the most electronegative elements none of these concepts can adequately describe the bond.^[2]^ Triatomic molecules lead us to the simplest cases of multi‐centre bonds and the trihalogen monoanions [A_3_]^−^ (X=F, Cl, Br, I)^[3]^ are the simplest representatives featuring a so‐called halogen bond interaction,[^4^, ^5^, ^6^, ^7^] a rapidly growing field in chemistry^[8]^ and material science^[9]^ that currently creates a wide range of applications.^[10]^ The bonding in these molecules is usually described in terms of a simplified (3c–4e) σ molecular orbital (MO) bond model, restricted to the three‐axial *n*pσ orbitals, which form one bonding (1σ_u_^+^) and a nonbonding (1σ_g_^+^) MO.[^11^, ^12^, ^13^] Alternatively, in the valence bond (VB) theory,[^14^, ^15^, ^16^] the bonding in trihalogen monoanions [A_3_]^−^ is described by a resonance of mainly four VB structures [Eq. 1].[^15^, ^16^, ^17^]

(1) $$A{}_{3}{}^{-}=A{}^{-}\;A\text{-}A\;\left(I\right)↔A\text{-}A\;A{}^{-}\;\left(\mathrm{II}\right)↔A{}^{-}\;A{}^{+}\;A{}^{-}\;\left(\mathrm{III}\right)↔A{}^{•}\;A{}^{-}\;A{}^{•}\;\left(\mathrm{IV}\right)$$

Since structures (I) and (II) differ from (IV) by a single electron transfer, mixing of these structures gives rise to a so‐called “charge‐shift resonance energy”,^[2]^ which was found to be the dominant contribution to the total bond dissociation energy.^[17]^ Hence, these σ‐type bonds were classified as “charge‐shift” (CS) bonds.^[2]^ According to a topological analysis of the computed electron density (ED) based on the quantum theory of atoms in molecules (QTAIM),[^18^, ^19^] or the electron localization function (ELF),^[18]^ CS bonds are characterized by high electron delocalization and fluctuation indices, respectively.[^2^, ^20^]

Herein, we present the synthesis and high‐resolution X‐ray structure analysis of a symmetric trichlorine monoanion [Cl_3_]^−^ in the tetrapropyl ammonium trichloride salt [N(*n*Pr)_4_][Cl_3_], **3**. A highly symmetric [Cl_3_]^−^ monoanion has so far only been observed in a matrix‐isolation study^[21]^ but not in bulk material. The high‐resolution X‐ray data from **3** enabled us to conduct the first topological analysis of an experimental charge‐density of a 22 valence electron system. This analysis not only allows a comparison of a quantum‐chemically computed ED with an experimental one from the crystalline state as probed by XRD, it also provides an insight into intermolecular interactions posed by the crystalline environment. In addition, the high‐resolution X‐ray structure and charge density analysis for two asymmetric trichlorine monoanions [NMe_3_EtCl][Cl_3_], **1**, and [NMe_4_][Cl_3_], **2**, provide insight into the influence of the crystalline environment on the Cl^−^⋅⋅⋅Cl_2_ interaction as well as into the progress of the bond formation between a dichlorine molecule and a Cl^−^ anion as visualized by the experimental ED of the reactants (Figure 1).

**Figure 1:**
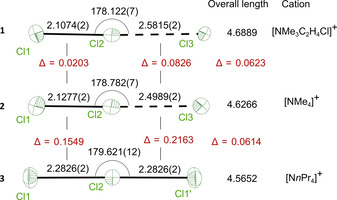
Anisotropic displacement parameters of the trichlorine ion of compounds **1**, **2**, and **3** together with the main metric and bond lengths differences *Δ* in [Å] and [°], respectively.^[31]^

**1** and **2** crystallize in the orthorhombic space group *Pnma*. The asymmetric unit contains only half the formula unit, hence many atoms, including all chlorine atoms, are located on the mirror plane.

In contrast, **3** crystallizes in the monoclinic space group *P*2/*n*. Here the asymmetric unit consists of only half of the molecule. However, the middle Cl atom of the trichlorine ion and the central N atom of the counter ion reside on a two‐fold axis leading to two identical Cl–Cl distances. Selected crystallographic data for all three compounds are listed in Table S1. A comparative overview of the geometry of all three trichlorine anions is depicted in Figure 1. Overall, the geometry of all three trichlorine anions is close to linear. As a general trend, shortening the Cl1−Cl2 bond from **3** to **1** elongates the Cl2⋅⋅⋅Cl3 bond, while the overall total length of the [Cl_3_]^−^ ion increases by about 0.12 Å, although all high‐resolution data sets were collected at the same temperature.

Table S23 lists the most important QTAIM[^19^, ^22^, ^23^] parameters for the trichlorine anions of **1**–**3** and theoretical values obtained by density functional theory (DFT) calculations at the B3LYP/def2‐TZVP level for periodic crystalline solids and isolated [Cl_3_]^−^ and Cl_2_ molecules. The electron density at the bond critical point *ρ*(*r*_BCP_) of the Cl−Cl bonds ranges from 0.27 to 0.84 e Å^−3^. The shorter Cl1−Cl2 bond in **1** and **2** has approximately twice the electron density of the longer Cl2⋅⋅⋅Cl3 bond, while the Cl–Cl distances of **3** show, as expected, an intermediate *ρ*(*r*_BCP_) value of 0.6 e Å^−3^. The calculated *ρ*(*r*_BCP_) values agree well with the experimental ones and are consistent with those for isolated [Cl_3_]^−^, which decay exponentially with the bond lengths (Figure 2). For the ∇^2^ *ρ*(*r*_BCP_), the calculated values increase for short bond lengths, reach a maximum for the equilibrium bond length of [Cl_3_]^−^, and decay again for larger bond lengths. The experimentally determined ∇^2^ *ρ*(*r*_BCP_) values deviate from this trend for short bonds in **1** and **2**, as known also from other experimental measurements.^[24]^

**Figure 2:**
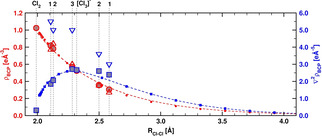
Calculated and experimentally determined electron density [*ρ*(*r*_BCP_), red] and Laplacian [∇^2^ *ρ*(*r*_BCP_), blue] at the bond critical points (BCP) with respect to the Cl–Cl distance, *R*_Cl–Cl_. The red and blue line and small symbols show values for isolated [Cl_3_]^−^ with different bond distances (see SI for further details). Large red circles show calculated values for *ρ*(*r*_BCP_), large blue squares show calculated values for ∇^2^ *ρ*(*r*_BCP_), and triangles correspond to measured values for systems **1**–**3**. The vertical lines mark the bond lengths present in isolated Cl_2_, [Cl_3_]^−^, and the systems **1**–**3**. A relatively small *ρ*(*r*_BCP_) and a positive Laplacian ∇^2^ *ρ*(*r*_BCP_) point to a serious electron density depletion at the BCP.

Consistent with the VB description [Eq. (1)] the Cl−Cl bonds are predominantly ionic with minute covalent contributions. This is indicated by the values for *ρ*(*r*_BCP_) as well as the negative total electronic energy density *H*(*r*_BCP_) and the ratio of potential to kinetic energy density (|*V*|(*r*_BCP_)/ *G*(*r*_BCP_)).^[22]^ As expected, the shortest bonds, Cl1−Cl2 in **1** and **2**, show the highest ratio, although it still belongs to the intermediate covalent/ionic range. Also the ELF_BCP_ values confirm that the bonds explored here have minimal covalent content. In symmetric [Cl_3_]^−^ the electrons appear to be as delocalized as in a homogeneous electron gas of equal density (ELF_BCP_ value of about 0.5). When elongating one bond and shortening the other, the ELF_BCP_ value for the former decreases further—indicating the ionic character—while the ELF_BCP_ value of the latter increases, approaching the value in Cl_2_. However, even in dichlorine, ELF_BCP_ is much smaller than 1.0, reflecting its charge shift character.

The courses of the Laplacian and the electron density along the bond path for Cl1−Cl2 and Cl2⋅⋅⋅Cl3 are shown in Figure S20. In both bonds, the BCP is shifted towards the central Cl2, which is even more pronounced in the longer Cl2⋅⋅⋅Cl3 bond. Figure 3 shows the experimental (left) and the calculated (middle) Laplacians in a plane through the three chlorine atoms in structures **1**–**3**. As expected for an ionic interaction, the valence shell of Cl3 in **1** and **2** is much less polarized towards Cl2 than that of Cl1 or that in **3**. The valence‐shell charge concentration (VSCC) at the atoms Cl2 and Cl3 in **1** and **2** is characteristic for an ion‐like interaction, although in compound **2**, a stronger polarization along the bond path indicates a slightly higher covalent content (Figure 3, left).

**Figure 3:**
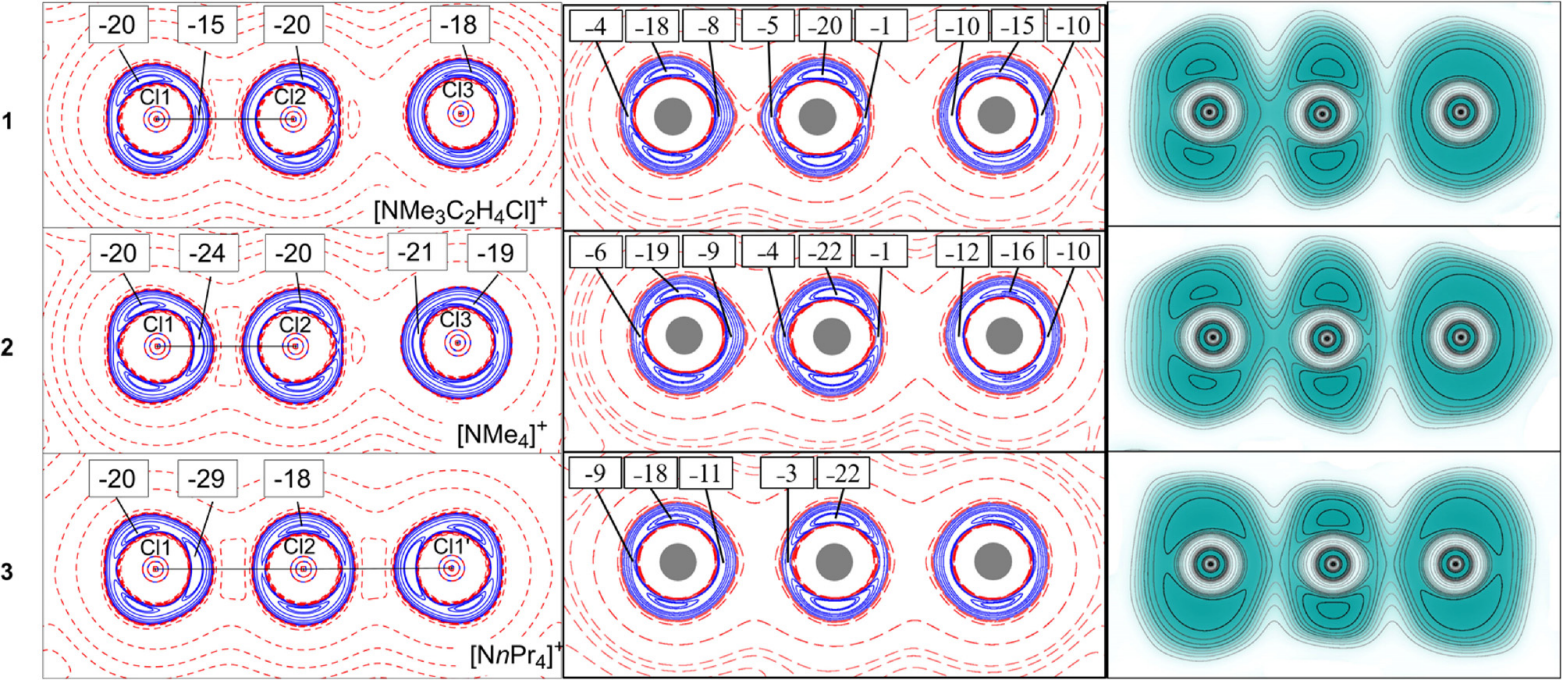
Experimental (left) and calculated (middle) Laplacian of the electron density and calculated ELF (right) for **1**–**3**. For the Laplacian, positive values are in red, negative values in blue, and contours are ±{0.001 0.002 0.004 0.008 0.02 0.04 0.08 0.2 0.4 0.8 2.0 4.0 8.0 14.0 16.0} e Å^−5^. The values of minima in the Laplacian are indicated. For ELF, values range from 0.0 (white) to 1.0 (dark cyan) with contours plotted from 0.1 to 1.0 in intervals of 0.1. The graphics at the left were created with XDGRAPH,^[25]^ the graphics in the middle and at the right were created with Gri.^[26]^

Qualitatively, the Laplacians from experimental and calculated electron densities agree very well, however, the former shows larger magnitudes at minima, predicting higher charge concentrations. These charge concentrations are slightly shifted from Cl2 towards Cl3 in the non‐equidistant trichlorine ions (see Figure S19), while in **3** they are located at the equator of Cl2. All three [Cl_3_]^−^ ions display a σ‐hole^[27]^ at the back side of Cl1. The non‐equidistant trichlorine anions **1** and **2** also show a σ‐hole at Cl2 in the direction to Cl3. Both Cl3 atoms adopt a relatively spherical charge distribution with no noticeable holes.

As in the Laplacian, the two lone pairs of Cl2 in the ELF plots move towards Cl3 in **1** and **2**, starting to resemble the ELF plot of isolated Cl_2_ (cf. Figure S22). But the two ELF maxima from the lone pairs of Cl3—unlike in the Laplacian—dissolve into a spherical area of high ELF value, precisely as one would expect for the valence shell of a chloride ion engaged in a non‐shared interaction. In all systems, the valence shell is clearly separated from the core region by a sphere of nearly zero ELF values. For all Cl1 (Cl1′) and Cl2, ELF shows two maxima (as the result of a torus cut in half) with ELF values close to 1.0, representing the lone pairs of these atoms. Between these atoms in **1** and **2**, ELF reaches values of about 0.65 (cf. Table S23). This is significantly lower than 1.0, which one would expect for a genuine covalent bond, reflecting these bonds’ charge‐shift nature. For the symmetric trichlorine ion **3**, ELF is again smaller, indicating a more delocalized character of the electrons in these bonds. Finally, for the longest bonds in **1** and **2**, ELF decreases further, indicating a vanishing covalent contribution. Previous studies on halogen−halogen bonding[^4^, ^6^, ^7^] and the discovery of large polychlorine anions, for example, [Cl_11_]^−^, [Cl_12_]^2−^, and [Cl_13_]^−^,^[28]^ encouraged us to look closer at intermolecular Cl⋅⋅⋅Cl interactions. However, even the shortest inter‐molecular Cl⋅⋅⋅Cl contacts, which are observed in **1**, exceed twice the van der Waals radius of chlorine, and the corresponding *ρ*(*r*_BCP_) values are too small (0.03 e Å^−3^) to be associated with a significant Cl−Cl interaction (cf. Table S21).

There is a significant number of C−H⋅⋅⋅halogen contacts with distances below the sum of the van der Waals radii, especially for **1** and **2**. In the solid‐state they are commonly[^12^, ^29^] rendered responsible for the deviation of the [A_3_]^−^ anions from equidistance. In Figure 4, these contacts between the [Cl_3_]^−^ ions and their related cations are illustrated by colour‐coded Hirshfeld surfaces.^[30]^ Areas in red indicate these short contacts (see Table S24); areas in blue those longer than the sum of the van der Waals radii. The differences between the non‐equidistant trichlorine ions in **1** and **2** and the equidistant ones in **3** are apparent. The shortest contacts (2.629 Å) are observed for the most asymmetric [Cl_3_]^−^ anion in **1**. In **2**, the [Cl_3_]^−^ anion is exposed to more short C−H⋅⋅⋅Halogen contacts, but they are on average 0.04 Å longer. Anyhow, in both systems, these contacts are sufficient to stabilize the—at least in vacuum—less stable asymmetric [Cl_3_]^−^ anion. The higher symmetry of **3** arises from even weaker interacting [N*n*Pr_4_]^+^ cations.

**Figure 4:**
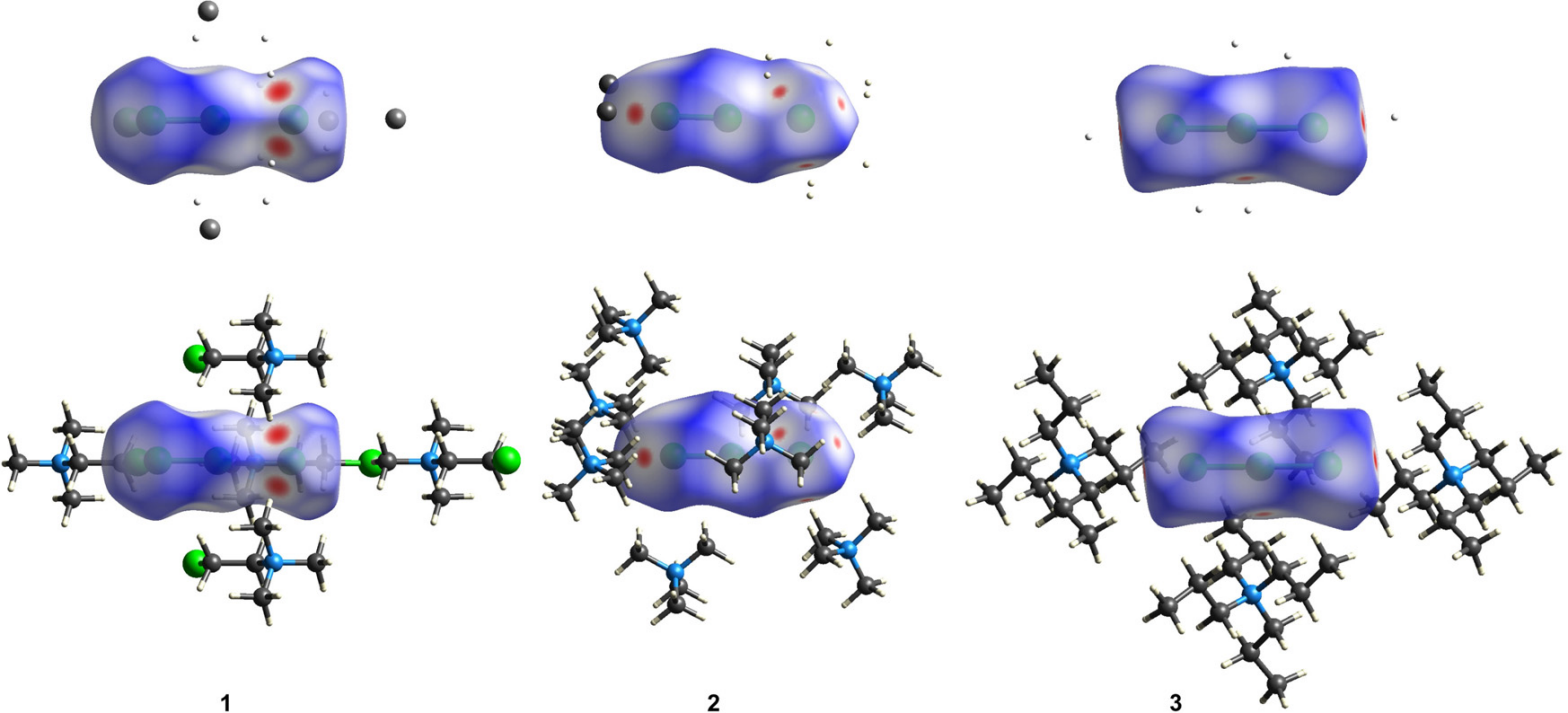
Hirshfeld surfaces^[30]^ of **1**–**3**. Surface colour codes for normalized contact distances: red: short, blue: long.

In summary, for the first time, the 3c–4e bond of a symmetric trichlorine anion was characterized by the topological analysis of the experimental density distribution and compared to two asymmetric trichlorine ions. Quantum‐chemical DFT calculations for both periodic crystals and isolated molecules/ions do not fully agree for the Laplacian but support the qualitative analysis of bond properties. There appears to be a smooth transition from the asymmetric to the symmetric compound. For the asymmetric trichlorine ions, short Cl⋅⋅⋅H contacts were detected, contributing to the considerable structural differences of these trichlorine anions.

## Conflict of interest

The authors declare no conflict of interest.

## Supporting information

As a service to our authors and readers, this journal provides supporting information supplied by the authors. Such materials are peer reviewed and may be re‐organized for online delivery, but are not copy‐edited or typeset. Technical support issues arising from supporting information (other than missing files) should be addressed to the authors.

Supplementary
