# Supplementary material for: Insights into the Topology and the Formation of a Genuine ppσ Bond: Experimental and Computed Electron Densities in Monoanionic Trichlorine [Cl3]−
Source: Angew Chem Int Ed Engl. 2020 Dec 1;60(5):2569–73. doi: 10.1002/anie.202013727 (PMC7898528; doi:10.1002/anie.202013727)
Supplement: Supplementary file 1 — Supplementary [file ANIE-60-2569-s001.pdf]

Angewandte Chemie
Eine Zeitschrift der Gesellschaft Deutscher Chemiker

Supporting Information

Insights into the Topology and the Formation of a Genuine ppσ Bond: Experimental and Computed Electron Densities in Monoanionic Trichlorine $[Cl_3]^-$

Helena Keil[+], Karsten Sonnenberg[+], Carsten Müller, Regine Herbst-Irmer, Helmut Beckers, Sebastian Riedel, and Dietmar Stalke**

anie_202013727_sm_miscellaneous_information.pdf

Supporting Information

Table of Contens

Table of Contens ... 1

S1. Synthesis and characterization of 1-3. ... 2

S1.1. 2-chloroethyltrimethylammonium trichloride $[NMe_3EtCl][Cl_3]$ (1) ... 2

S1.2. Tetramethylammonium trichloride $[NMe_4][Cl_3]$ (2) ... 2

S1.3. Tetrapropylammonium trichloride $[N\mathit{n}Pr_4][Cl_3]$ (3) ... 2

S2. X-ray experiment ... 2

S2.1. Data collection and handling ... 2

S2.2. Multipole refinement ... 3

S2.3. Crystallographic data for 1, 2 and 3 ... 3

S2.3.1. Multipole strategy and validation for 1 ... 3

S2.3.2. Cross-validation[13] ... 5

S2.3.3. Data quality[14]–[17] ... 6

S2.3.4. Refinement of anharmonic motion[18],[19] ... 7

S2.4. Multipole strategy and validation for 2 ... 8

S2.4.1. Cross-validation[13] ... 9

S2.4.2. Data quality[14]–[17] ... 10

S2.4.3. Refinement of anharmonic motion[18],[19] ... 11

S2.5. Multipole strategy and validation for 3 ... 12

S2.5.1. Cross-validation[13] ... 14

S2.5.2. Data quality[13]–[16] ... 14

S2.5.3. Refinement of anharmonic motion[18],[19] ... 15

S2.5.4. Properties ... 17

S2.5.5. QTAIM parameters for 1 ... 19

S2.5.6. QTAIM parameters for 2 ... 20

S2.5.7. QTAIM parameters for 3 ... 20

S3. Laplacian and ELF for isolated Cl_2 and $[Cl_3]^-$ ... 21

S4. Computational Details ... 21

S5. References ... 21

S1. Synthesis and characterization of 1-3.

All preparative work was carried out using standard Schlenk-techniques. Chlorine (Linde, purity 2.8) was passed through calcium chloride to remove traces of water. Tetramethylammonium chloride, tetrapropylammonium chloride and 2-chloroethyltrimethylammonium chloride were dried for 2 days at reduced pressure at 60°C. Raman spectra were recorded on a Bruker (Karlsruhe, Germany) MultiRAM II equipped with a low-temperature Ge detector (1064 nm, 100 mW, resolution 4 cm–1). Single-crystal Raman spectra of cooled, crystalline samples were measured with a Bruker RamanScope III. NMR spectra were obtained on a JEOL ECS 400 NMR spectrometer at room temperature.

S1.1. 2-chloroethyltrimethylammonium trichloride [NMe_3EtCl][Cl_3] (1)

2-chloroethyltrimethylammonium chloride (691 mg, 4.4 mmol, 1.0 eq) was dissolved in acetonitrile (2 mL) and chlorine (312 mg, 4.4 mmol, 1.0 eq) was condensed onto the solution and warmed up to room temperature. The yellow solution was slowly cooled to –40°C and crystals were obtained.
1H-NMR (400 MHz, 25°C, D2O): δ = 4.01 (t, 2H, CH2CH2Cl), 3.77 (t, 2H, NCH2CH2), 3.21 (a, 9H, NCH3) ppm.
Raman (77 K): 3029 (w), 2985 (w), 2959 (w), 1444 (w), 1385 (vw), 779 (m), 736 (w), 370 (s), 351 (vs), 279 (vw), 137 (vw), 114 (w) cm–1.

S1.2. Tetramethylammonium trichloride [NMe_4][Cl_3] (2)

Tetramethylammonium chloride (300 mg, 2.7 mmol, 1.0 eq) was dissolved in acetonitrile (1.2 mL) and gaseous chlorine was passed through the suspension until a clear yellow solution was obtained. The solution was cooled to 2°C and crystals were obtained.
1H-NMR (400 MHz, 21°C, D2O): δ = 3.17 (s, 12H, NCH3) ppm.
Raman (77 K): 3022 (m), 2978 (vw), 2952 (w), 2918 (w), 2810 (vw), 1487 (vw), 1459 (vw), 1413 (vw), 1291 (vw), 949 (vw), 753 (vw), 528 (vw), 459 (vw), 344 (vs), 314 (m), 173 (sh, vw), 161 (w) cm–1.

S1.3. Tetrapropylammonium trichloride [N*n*Pr_4][Cl_3] (3)

Tetrapropylammonium chloride (126 mg, 1.8 mmol, 1.0 eq) was dissolved in acetonitrile (1.5 mL) and gaseous chlorine was passed through the suspension until a clear yellow solution was obtained. The solution was cooled to –20°C and crystals were obtained.
1H-NMR (400 MHz, 21°C, CDCl3): δ = 3.34 (t, 8H, NCH2CH2), 1.76 (m, 8H, CH2CH2CH3), 1.05 (t, 12H, CH2CH3) ppm.
Raman (77 K): 2999 (w), 2974 (w), 2954 (w), 2930 (w), 2911 (w), 2879 (w), 1454 (w), 366 (vw), 314 (vw), 270 (vs), 125 (vw) cm–1.

S2. X-ray experiment

S2.1. Data collection and handling

High resolution X-ray diffraction data of **1** and **2** were collected on a Bruker SMART APEX II diffractometer based on D8 three-circle goniometer system using an Incoatec microfocus MoKα source (IμS) and Incoatec QUAZAR mirror optics.[1] High resolution single X-ray data of **3** was collected on a Bruker SRA TXS rotating anode with molybdenum as anode material. A Bruker APEX II CCD detector was used to record the diffracted intensities at λ = 0.71073 Å. The single crystals were mounted form inert oil at low temperature and under nitrogen atmosphere using the X-Temp 2 device[2]. Data reduction was carried out with SAINT[3] (version 8.38A) from the APEX3[4] program package in which the integration box sizes were refined for every run using a standard procedure for **1**. For **2** and **3** the integration box was fixed to [0.7 0.75 0.4] and [0.7 0.7 0.4], respectively. The data were scaled, equivalent reflections were merged and an empirical absorption correction was performed with SADABS[5] (version 2016/2). Afterwards, the structure was solved with SHELXT[6] using direct methods and refined by full-matrix least square against F^2 using SHELXL[7] (version 2018/1) by means of the graphical user interface SHELXle[8].

S2.2. Multipole refinement

The multipole refinement using the nucleus-centred multipole model of Hansen & Coppens[9] was carried out on F^2 with the full-matrix-least-squares refinement program XDLSM implemented in the XD2016 (version 2016/1) program[10]. Topological analysis according to the Quantum Theory of Atoms in Molecules[11] (QTAIM) was performed using the XDPROP and TOPXD programs included in the XD package.

S2.3. Crystallographic data for 1, 2 and 3

Table S1. Crystallographic details at 100 K.

Compound	**1**	**2**	**3**
CCDC number	2030908	2030909	2030910
Formula	$[NMe_3C_2H_4Cl][Cl_3]$	$[NMe_4][Cl_3]$	$[N\mathit{n}Pr_4][Cl_3]$
λ (Å)	0.71073	0.71073	0.71073
Crystal system,	Orthorhombic	Orthorhombic	Monoclinic
Space group	*Pnma*	*Pnma*	*P*2/*n*
a (Å)	14.100(3)	10.412(2)	8.511(2)
b (Å)	7.128(2)	7.681(2)	7.253(2)
c (Å)	10.106(2)	11.432(3)	12.970(3)
β (°)	90	90	92.33(2)
Crystal size (mm)	0.303 x 0.247 x 0.226	0.308 x 0.246 x 0.198	0.150 x 0.250 x 0.290
Volume (Å^3)	1015.7(4)	914.3(4)	800.0(3)
Z	4	4	2
Density (Mg/m^3)	1.497	1.311	1.215
μ (mm^{-1})	1.102	0.922	0.552
F(000)	472	376	316
$(\sin\theta/\lambda)_{min}$, $(\sin\theta/\lambda)_{max}$ (Å^{-1})	0.061, 1.137	0.065, 1.111	0.069, 1.066
Ref. meas., Ref. unique	67348, 6534	109717, 5489	179675, 8103
R_{int}	0.0253	0.0237	0.0446
**IAM Refinement**			
Data / restraints / parameters	6534 / 0 / 59	5489 / 0 / 47	8103 / 0 / 76
Final R indices [I>2σ(I)]	R1 = 0.0191,	R1 = 0.0216	R1 = 0.0275
	wR2 = 0.0542	wR2 = 0.0657	wR2 = 0.0759
R indices (all data)	R1 = 0.0223,	R1 = 0.0252	R1 = 0.0377
	wR2 = 0.0561	wR2 = 0.0692	wR2 = 0.0830
$\Delta\rho_{max}$, $\Delta\rho_{min}$ (e Å^{-3})	0.819, -0.976	0.673, -0.727	0.653, -0.192
**Multipole Refinement Experimental Data**			
Data / parameters	6337/ 146	5233/ 132	7464/ 145
$R_1(F^2)$	0.0139	0.0141	0.0200
GOF	1.451	1.907	2.0350
$\Delta\rho_{max}$, $\Delta\rho_{min}$ (e Å^{-3})	0.182, -0.216	0.432, -0.193	0.358, -0.244

S2.3.1. Multipole strategy and validation for 1

Table S2. Local coordinate system.

ATOM	ATOM0	AX1	ATOM1	ATOM2	AX2	KAP	SITESYM	CHEMCON
Cl(1)	Cl(2)	Z	Cl(1)	DUM8	Y	1	cyl	
Cl(2)	Cl(1)	Z	Cl(2)	DUM7	X	1	cyl	
Cl(3)	Cl(2)	Z	Cl(3)	DUM6	Y	1	cyl	
Cl(4)	C(4)	Z	Cl(4)	DUM0	Y	5	cyl	
N(1)	Cl(1)	Z	N(1)	DUM3	Y	2	mY3Z	
C(1)	N(1)	Z	C(1)	H(1Y)	Y	3	mY3Z	
C(2)	N(1)	Z	C(2)	H(2Y)	Y	3	mX3Z	
C(3)	N(1)	Z	C(3)	DUM2	Y	3	mY	
C(4)	Cl(4)	Z	C(4)	DUM1	Y	3	mY	
H(2X)	C(2)	Z	H(2X)	H(2Z)	Y	4	cyl	H(2X)
H(2Y)	C(2)	Z	H(2Y)	H(2Z)	Y	4	cyl	H(2X)
H(2Z)	C(2)	Z	H(2Z)	H(2X)	Y	4	cyl	H(2X)
H(3)	C(3)	Z	H(3)	N(1)	Y	4	cyl	H(2X)

H(4)	C(4)	Z	H(4)	C(3)	Y	4	cyl	H(2X)
H(1X)	C(1)	Z	H(1X)	H(1Y)	Y	4	cyl	H(2X)
H(1Y)	C(1)	Z	H(1Y)	DUM5	Y	4	cyl	

Monopole and multipole parameters of atoms C(1) and H(1Y) were constraint to C(2) and H(2X), respectively.

Table S3. Fractional coordinates for dummy atoms.

DUM	x	y	z
0	0.093296	-0.25	0.460205
1	0.177460	-0.25	0.327204
2	0.275367	-0.25	0.389751
3	0.355924	-0.25	0.291272
4	0.446080	-0.25	0.370343
5	0.497100	-0.25	0.310900
6	0.658164	-0.25	0.517574
7	0.815827	-0.25	0.646287
8	0.942580	-0.25	0.757446

Table S4. The following table in combination with the cross-validation[12] plots in Figure S1 and S2 illustrates which parameters can be refined without any indication of overfitting. The last four steps have been omitted as they lead to over-fitting. Abbreviations: Sf: scale factor; MP: multipoles; M: monopoles; D: dipoles; Q: quadrupoles; O: octupoles; H: hexadecapoles, U: Uij, U3: third order Gram-Charlier coefficient, k: kappa, k': kappa prime, GOF. Goodness of Fit; low-res data: data < 0.5 sin(Θ)/λ. Every new added parameter is marked in red.

Step	Refined parameter	Data	MP-param.	Ratio low-res data to MP, k & k'	Param.	Ratio data to param.	GOF	$R1(F^2)$
1	Sf	6337	0	0	1	6337	4.602	0.0334
2	Sf, DQO	6337	35	16.9	41	154.6	3.034	0.0234
3	Sf, MDQO	6337	44	13.4	49	129.3	2.675	0.0219
4	Sf, MDQO, U	6337	44	13.4	87	72.8	2.447	0.0209
5	Sf, MDQO, U3 (Cl1, Cl2, Cl3, Cl4)	6337	44	13.4	111	57.1	2.094	0.0185
6	Sf, MDQO, U, U3, XYZ	6337	44	13.4	130	48.8	1.923	0.0173
7	Sf, MDQO, U, U3, XYZ, k	6337	44	12.3	134	47.3	1.831	0.0167
8	Sf, H-XYZ	590	0	0	21	28.1	3.954	0.0154
9	Sf, MDQO, U, U3, XYZ, k	6337	44	12.3	134	47.3	1.65	0.0154
10	Sf, k'	6337	4	147.5	5	1267.4	1.592	0.0151
11	Sf, MDQO, U, U3, XYZ, k	6337	44	12.3	134	47.3	1.574	0.0149
12	Sf, MDQOH(all C-atoms), U, U3, XYZ, k	6337	53	10.3	146	43.4	1.502	0.0143
13	Sf, MDQOH(all Cl atoms), U, U3, XYZ, k	6337	57	9.7	150	42.2	1.498	0.0143
14	Sf, MDQOH(N1), U, U3, XYZ, k	6337	58	9.5	151	42	1.497	0.0143
15	Sf, MDQOH, U, U3, XYZ, k, cyl > mm2 (all Cl atoms)	6337	66	8.4	159	39.9	1.496	0.0143
16	Sf, MDQOH, U, U3, XYZ, k, mm2 > m (all Cl atoms)	6337	82	6.9	175	36.2	1.479	0.0141

Table S5. Final refinement.

Step	Refined parameter	Data	MP-param.	Ratio low-res data to MP, k & k'	Param.	Ratio data to param.	GOF	R(F2)
1-11	Parameters 1-12 from table S4	6337	53	10.3	146	43.4	1.477	0.0141
12	k‘	6337	4	147.5	5	1267.4	1.445	0.0139
13	Sf, MDQOH(all C-atoms), U, U3, XYZ,	6337	53	10.3	146	43.4	1.451	0.0139

S2.3.2. Cross-validation[13]

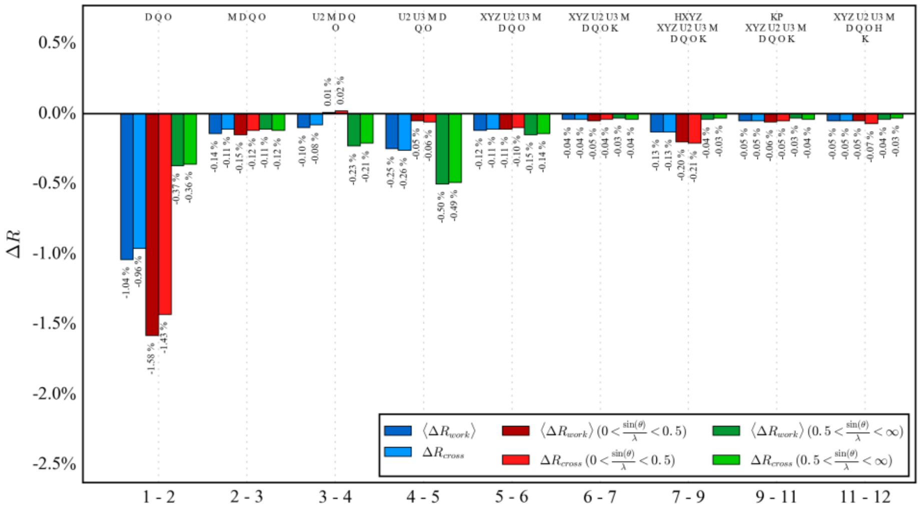

Figure S1. Cross-validation plot for steps 1-12.

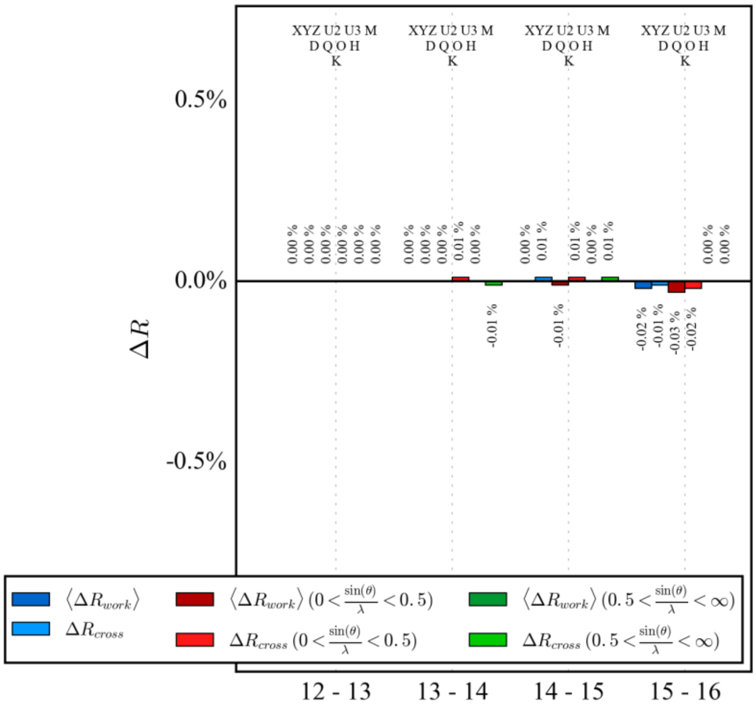

Figure S2. Cross-validation plot for steps 12-16.

S2.3.3. Data quality[14]–[17]

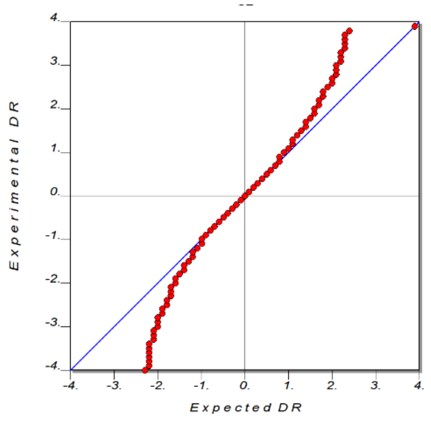

Figure S3. **Normal probability plot.**

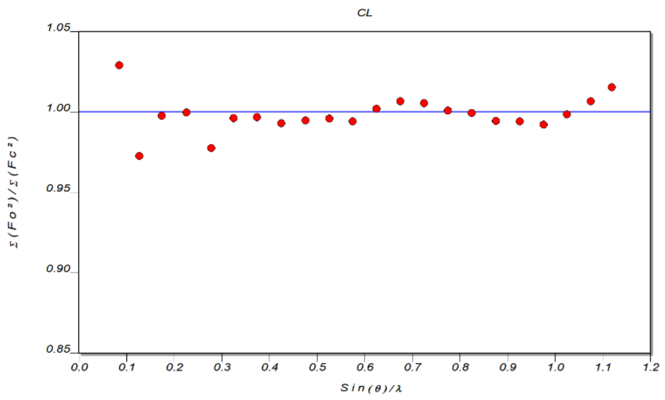

Figure S4. **DRK plot.**

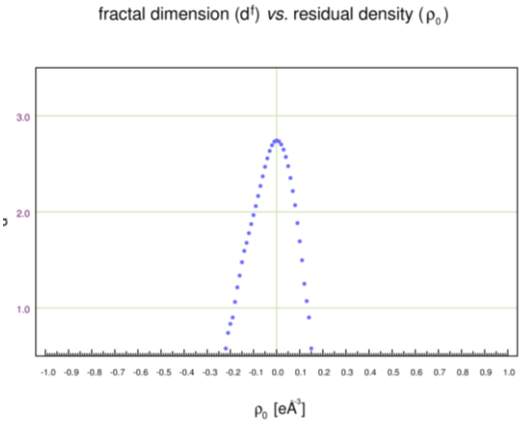

Figure S5. **Henn-Meindl plot.**

S2.3.4. Refinement of anharmonic motion[18],[19]

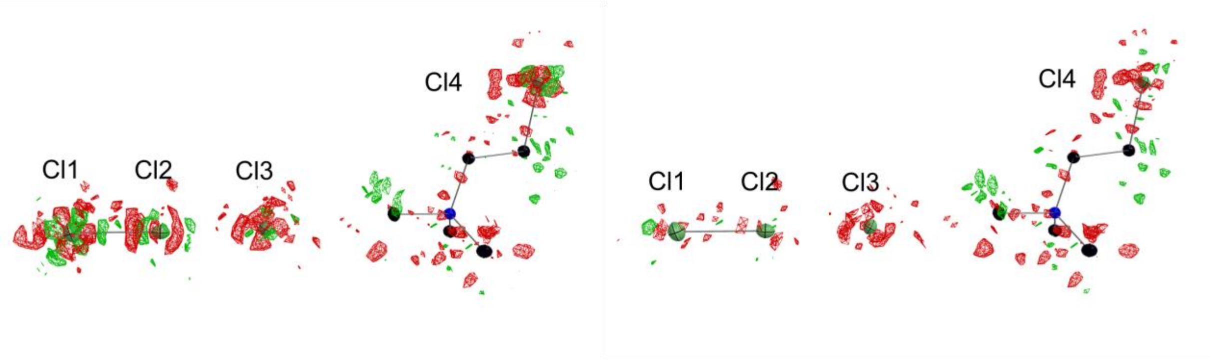

Figure S6. Residual density before and after anharmonic refinement. The isosurface value is ±0.08 eÅ^{-3}. Positive contours are plotted with green lines and negative contours are plotted with red lines. The graphics were created with MoleCoolQT[19].

Table S6. The table below shows the minimum data resolution required for meaningful refinement of anharmonic thermal parameters (Gram-Charlier coefficients), for each anisotropic atom.[20]

Atom	Principal M.D.A's (A)			Min. resolution Qn sin(theta)/lambda	
				n =3	n = 4
Cl1	0.164	0.158	0.134	0.86	0.99
Cl2	0.153	0.122	0.110	1.02	1.17
Cl3	0.133	0.132	0.112	1.03	1.19
Cl4	0.143	0.131	0.103	1.04	1.20

Table S7. Calculated vibrational probability density function.

Atom	Total integrated negative probability [%]	Total integrated positive probability[%]	Maximum PDF value	Minimum PDF value	Integrated volume for negative probability [Å^3]	Integrated volume for positive probability [Å^3]
Cl1	-0.006	100.006	18573.34	-6.12	1.016	3.320
Cl2	-0.001	100.001	31294.84	-0.52	0.717	3.619
Cl3	0.000	100.000	32783.31	-0.02	0.702	3.633
Cl4	0.000	100.000	33494.86	-0.42	1.016	3.319

Table S8. Hirshfeld-test[21] after final refinement step. Differences of the mean-square displacement amplitudes (DMSDA) (1 × 10-4 Å^2) along atomic vectors.

ATOM	ATOM	DIST	DMSDA
Cl1	Cl2	2.1074	44
Cl2	Cl3	2.5815	-94
Cl4	C4	1.7937	4
N1	C1	1.5015	3
N1	C2	1.5013	4
N1	C3	1.5115	2
C3	C4	1.5184	3

In the case of strongly polar/ionic bonds, the rigid-bonds model is not fulfilled.

S2.4. Multipole strategy and validation for 2

Table S9. Local coordinate system.

ATOM	ATOM0	AX1	ATOM1	ATOM2	AX2	KAP	SITESYM	CHEMCON
Cl(1)	Cl(2)	Z	Cl(1)	DUM2	Y	1	cyl	
Cl(2)	Cl(1)	Z	Cl(2)	DUM1	Y	1	cyl	
Cl(3)	Cl(2)	Z	Cl(3)	DUM0	Y	1	cyl	
N(1)	C(3)	Z	N(1)	DUM5	Y	2	mY3Z	
C(1)	N(1)	Z	C(1)	H(1X)	Y	3	mX3Z	
C(2)	N(1)	Z	C(2)	H(2Y)	Y	3	mY3Z	
C(3)	N(1)	Z	C(3)	H(3X)	Y	3	mY3Z	C(2)
H(1X)	C(1)	Z	H(1X)	H(1Y)	Y	4	cyl	
H(1Y)	C(1)	Z	H(1Y)	H(1X)	Y	4	cyl	H(1X)
H(1Z)	C(1)	Z	H(1Z)	H(1Y)	Y	4	cyl	H(1X)
H(2X)	C(2)	Z	H(2X)	H(2Y)	Y	4	cyl	H(1X)
H(2Y)	C(2)	Z	H(2Y)	DUM6	Y	4	cyl	
H(3X)	C(3)	Z	H(3X)	DUM7	Y	4	cyl	H(2Y)
H(3Y)	C(3)	Z	H(3Y)	H(3X)	Y	4	cyl	H(1X)

The constraining of all H and C atoms, respectively, leads to worsening of the refinement in terms of residual density and R value.

Table S10. Fractional coordinates for dummy atoms.

DUM	x	y	z
0	0.20313	1.75	0.57936
1	0.38198	1.75	0.43339
2	0.53141	1.75	0.30609
3	0.82580	1.25	0.09519
4	0.63928	1.25	-0.03588
5	0.68205	1.25	0.08947
6	0.85340	1.25	0.17060
7	0.55370	1.25	-0.03630

Table S11. The following table in combination with the cross-validation[12] plots in Figure S7 and S8 illustrates which parameters can be refined without any indication of over-fitting. The last three steps have been omitted as they lead to over-fitting. Abbreviations: Sf: scale factor; MP: multipoles; M: monopoles; D: dipoles; Q: quadrupoles; O: octupoles; H: hexadecapoles, U: Uij, U3: third order Gram-Charlier coefficient, k: kappa, k': kappa prime, GOF. Goodness of Fit; low-res data: data < 0.5 sin(Θ)/l. Every new added parameter is marked in red.

Step	Refined parameter	Data	MP-param.	Ratio low-res data to MP, k & k'	Param.	Ratio data to param.	GOF	$R(F^2)$
1	Sf	5233	0	0	1	5233	6.452	0.0359
2	Sf, DQO	5233	25	21	26	201.3	4.353	0.0286
3	Sf, MDQO	5233	33	15.9	33	158.6	4.16	0.0286
4	Sf, MDQO, U	5233	33	15.9	63	83.1	3.351	0.0205
5	Sf, MDQO, U3 (Cl1, Cl2, Cl3, Cl4)	5233	33	15.9	81	64.6	2.597	0.0176
6	Sf, MDQO, U3, U4 (Cl1, Cl2, Cl3, Cl4)	5233	33	15.9	108	48.5	2.446	0.0169
7	Sf, MDQO, U, U3, U4, XYZ	5233	33	15.9	123	42.5	2.187	0.0157
8	Sf, MDQO, U, U4, XYZ, k	5233	33	14.6	126	41.5	2.149	0.0154
9	Sf, k'	5233	3	174.7	4	1308.2	2.096	0.0152
10	Sf, MDQO, U, U3, U4, XYZ, k	5233	33	14.6	126	41.5	2.098	0.0152
11	Sf, H-XYZ	524	0	0	20	26.2	4.443	0.0142

12	Sf, MDQO, U, U3, U4, XYZ, k	5233	33	14.6	126	41.5	1.984	0.0143
13	Sf, MDQOH(all C-atoms), U, U3, U4, XYZ, k	5233	37	13.1	130	40.2	1.918	0.0142
14	Sf, MDQOH(N1), U, U3, U4, XYZ, k	5233	39	12.5	132	39.6	1.908	0.0141
15	Sf, MDQOH(Cl atoms), U, U3, U4, XYZ, k	5233	42	11.6	135	38.8	1.905	0.0141
16	Sf, MDQOH, U, U3, U4, XYZ, k, cyl > mm2 (all Cl atoms)	5233	48	10.3	141	37.1	1.867	0.0139
17	Sf, MDQOH, U, U3, U4, XYZ, k, mm2 > m (all Cl atoms)	5233	52	9.5	145	36.1	1.838	0.0138

Table S12. Final Refinement.

Step	Refined parameter	Data	MP-param.	Ratio low-res data to MP, k & k'	Param.	Ratio data to param.	GOF	R(F2)
1-12	Parameters 1-14 from table S11	5233	39	12.5	132	5233	1.907	0.0141
13	k'	5233	3	174.7	4	1308.2	4.119	0.014
14	Sf, MDQOH(N1), U, U3, U4, XYZ, k	5233	39	12.5	132	5233	1.902	0.0141

S2.4.1. Cross-validation[13]

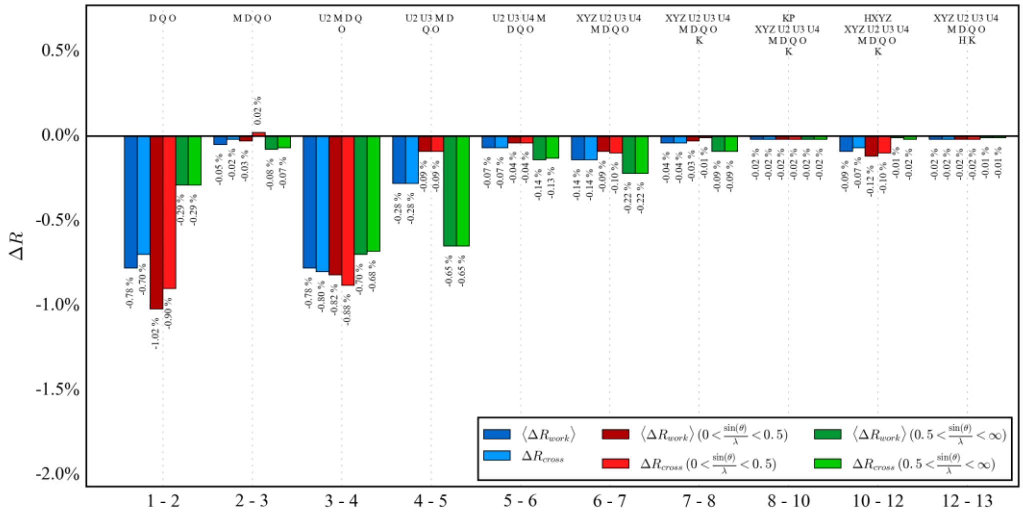

Figure S7. Cross-validation for Step 1-13.

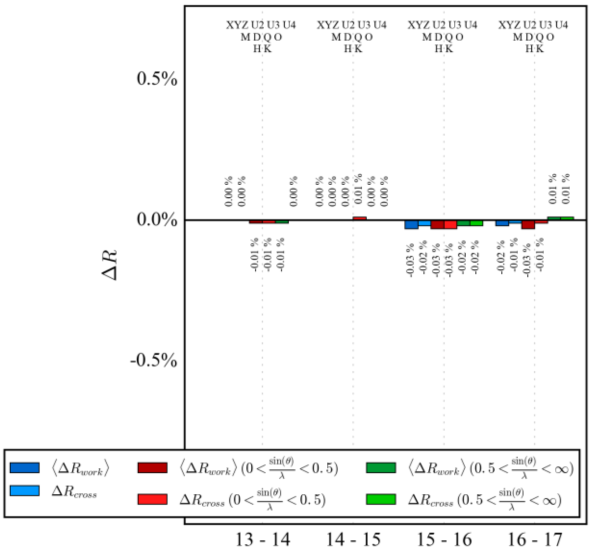

Figure S8. **Cross-validation for steps 13-17.**

Release of the local symmetry at the three Cl atoms (steps 16 and 17) improves the R value, but can only slightly reduce the residual electron density near the trichloride and therefore does not improve the model. Quite in contrary, it can be assumed that the trial to model this residual density with multipoles will distort the multipole parameters that are used to analyse the bonding situation. Additionally, we want to use the same refinement strategy for all three structures.

S2.4.2. Data quality[14]–[17]

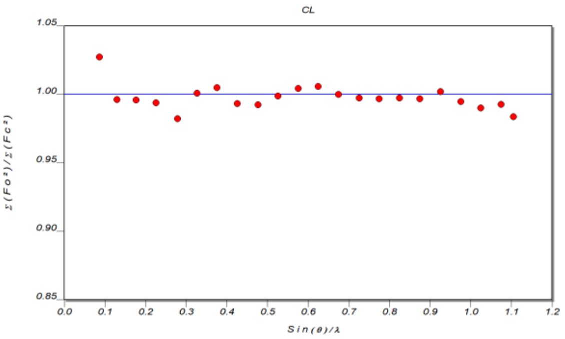

Figure S9. **DRK Plot.**

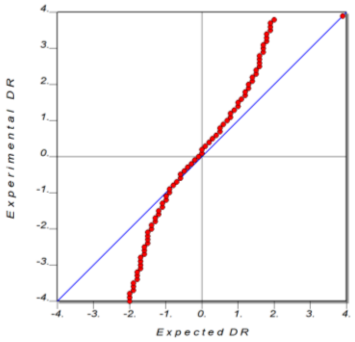

Figure S10. **Normal Probability Plot.**

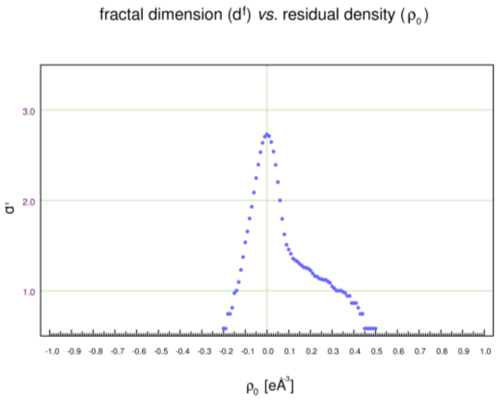

Figure S11. **Henn-Meindl plot.**

S2.4.3. Refinement of anharmonic motion[18],[19]

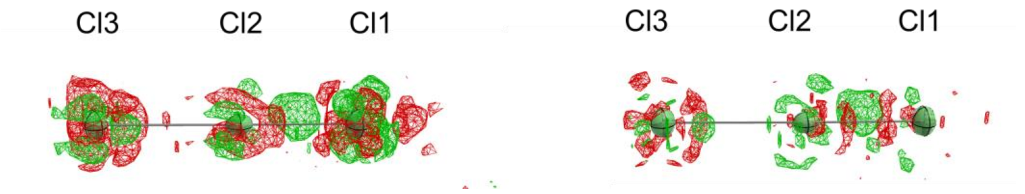

Figure S12. Residual density before and after anharmonic refinement. The isosurface value is ±0.06 eÅ^{-3}. Positive contours are plotted with green lines and negative contours are plotted with red lines. The graphics were created with MoleCoolQT[19].

Table S13. The table below shows the minimum data resolution required for meaningful refinement of anharmonic thermal parameters (Gram-Charlier coefficients), for each anisotropic atom.[20]

Atom	Principal M.D.A's (A)			Min. resolution Qn sin(theta)/lambda	
				n =3	n = 4
Cl(1)	0.182	0.178	0.148	0.77	0.89
Cl(2)	0.161	0.133	0.133	0.91	1.05
Cl(3)	0.176	0.148	0.142	0.84	0.97

Table S14. Calculated vibrational probability density function.

Atom	Total integrated negative probability [%]	Total integrated positive probability[%]	Maximum PDF value	Minimum PDF value	Integrated volume for negative probability [Å^3]	Integrated volume for positive probability [Å^3]
Cl(1)	-0.005	100.003	12233.69	-2.46	0.939	3.416
Cl(2)	-0.003	100.003	20123.07	-0.59	1.488	2.868
Cl(3)	-0.006	100.006	15287.49	-1.50	1.784	2.571

Table S15. Hirshfeld-test[21] after final refinement step. Differences of the mean-square displacement amplitudes (DMSDA) (1 × 10-4 Å2) along atomic vectors.

ATOM	ATOM	DIST	DMSDA
Cl(1)	Cl(2)	2.1277	32
Cl(2)	Cl(3)	2.4989	-42
N(1)	C(1)	1.4994	2
N(1)	C(2)	1.4982	3
N(1)	C(3)	1.4991	5

In the case of strongly polar/ionic bonds, the rigid-bonds model is not fulfilled.

S2.5. Multipole strategy and validation for 3

Table S16. Local coordinate system.

ATOM	ATOM0	AX1	ATOM1	ATOM2	AX2	KAP	SITESYM	CHEMCON
Cl(1)	Cl(2)	Z	Cl(1)	C(6)	Y	1	cyl	
Cl(2)	Cl(1)	Z	Cl(2)	DUM1	Y	1	cyl	
N(1)	DUM0	Z	N(1)	C(4)	Y	2	mX	
C(1)	N(1)	Z	C(1)	C(2)	Y	3	mX	
C(2)	C(1)	Z	C(2)	C(3)	Y	3	3ZmX	
C(3)	C(2)	Z	C(3)	H(3B)	Y	3	mX	
C(4)	N(1)	Z	C(4)	C(5)	Y	3	mX	C(1)
C(5)	C(4)	Z	C(5)	C(6)	Y	3	3ZmX	C(2)
C(6)	C(5)	Z	C(6)	H(6A)	Y	3	cyl	C(3)
H(1A)	C(1)	Z	H(1A)	H(1B)	Y	4	cyl	
H(1B)	C(1)	Z	H(1B)	H(1A)	Y	4	cyl	H(1A)
H(2A)	C(2)	Z	H(2A)	H(2B)	Y	4	cyl	H(1A)
H(2B)	C(2)	Z	H(2B)	H(2A)	Y	4	cyl	H(1A)
H(3A)	C(3)	Z	H(3A)	H(3B)	Y	4	cyl	
H(3B)	C(3)	Z	H(3B)	H(3C)	Y	4	cyl	H(3A)
H(3C)	C(3)	Z	H(3C)	H(3A)	Y	4	cyl	H(3A)
H(4A)	C(4)	Z	H(4A)	H(4B)	Y	4	cyl	H(1A)
H(4B)	C(4)	Z	H(4B)	H(4A)	Y	4	cyl	H(1A)
H(5A)	C(5)	Z	H(5A)	H(5B)	Y	4	cyl	H(1A)
H(5B)	C(5)	Z	H(5B)	H(5A)	Y	4	cyl	H(1A)
H(6A)	C(6)	Z	H(6A)	H(6B)	Y	4	cyl	H(3A)
H(6B)	C(6)	Z	H(6B)	H(6C)	Y	4	cyl	H(3A)
H(6C)	C(6)	Z	H(6C)	H(6A)	Y	4	mX	H(3A)

Table S17. Fractional coordinates for dummy atoms.

DUM	x	y	z
0	0.75000000	0.36190087	0.75000000
1	0.75000000	-0.628383	0.25000000

Table S18. The following table in combination with the cross-validation[12] plots in Figure S13 and S14 illustrates which parameters can be refined without any indication of over-fitting. The last three steps have been omitted as they lead to over-fitting. Abbreviations: Sf: scale factor; MP: multipoles; M: monopoles; D: dipoles; Q: quadrupoles; O: octupoles; H: hexadecapoles, U: Uij, U3: third order Gram-Charlier coefficient, k: kappa, k': kappa prime, GOF. Goodness of Fit; low-res data: data < 0.5 sin(Θ)/l. Every new added parameter is marked in red.

Step	Refined parameter	Data	MP-param.	Ratio low-res pata to MP, k & k'	Param.	Ratio data to param.	GOF	$R(F^2)$
1	Sf	7464	0	0	1	7464	5.503	0.0486
2	Sf, DQO	7464	35	23.9	36	207.3	3.828	0.0368
3	Sf, MDQO	7464	41	20.4	41	182.1	3.796	0.0362
4	Sf, MDQO, U	7464	43	19.4	93	80.3	2.694	0.0247
5	Sf, MDQO, U3 (Cl1, Cl2, Cl3, Cl4)	7464	43	19.4	107	69.8	2.499	0.0232
6	Sf, MDQO, U, U3, XYZ	7464	43	19.4	130	57.4	2.349	0.0221
7	Sf, MDQO, U, U3, XYZ, k	7464	43	18.2	133	56.1	2.335	0.0221
8	Sf, k'	7464	3	278.7	4	1866	2.283	0.0219
9	Sf, MDQO, U, U3, XYZ, k	7464	43	18.2	133	56.1	2.276	0.0217
10	Sf, H-XYZ	836	0	0	43	19.4	4.798	0.0208
11	Sf, MDQO, U, U3, XYZ, k	7464	43	18.2	133	56.1	2.111	0.0206
12	Sf, MDQOH(all C-atoms), U, U3, XYZ, k	7464	55	14.4	145	51.5	2.034	0.02
13	Sf, MDQOH(N1), U, U3, XYZ, k	7464	58	13.7	148	50.4	2.033	0.02
14	Sf, MDQOH(Cl1, Cl2), U, U3, XYZ, k	7464	60	13.3	150	49.8	1.991	0.0195
15	Sf, MDQOH, U, U3, XYZ, k, cyl > mm2 (Cl1), cyl > 2 (Cl2)	7464	68	11.8	158	47.2	1.98	0.0193

Although step 14 brings an improvement in the refinement, the hexadecapoles have not been refined to ensure comparability between the three structures. Additionally, the deformation density does not look reasonable.

Table S19. Final refinement.

Step	Refined parameter	Data	MP-param.	Ratio low-res data to MP, k & k'	Param.	Ratio data to param.	GOF	R(F2)
1-11	Parameters 1-12 from table S18	7464	55	14.4	145	51.5	2.035	0.02
12	k'	7464	3	278.7	4	1866	4.418	0.02
13	Sf, MDQOH(all C-atoms), U, U3, XYZ, k	7464	55	14.4	145	51.5	2.023	0.02

S2.5.1. Cross-validation[13]

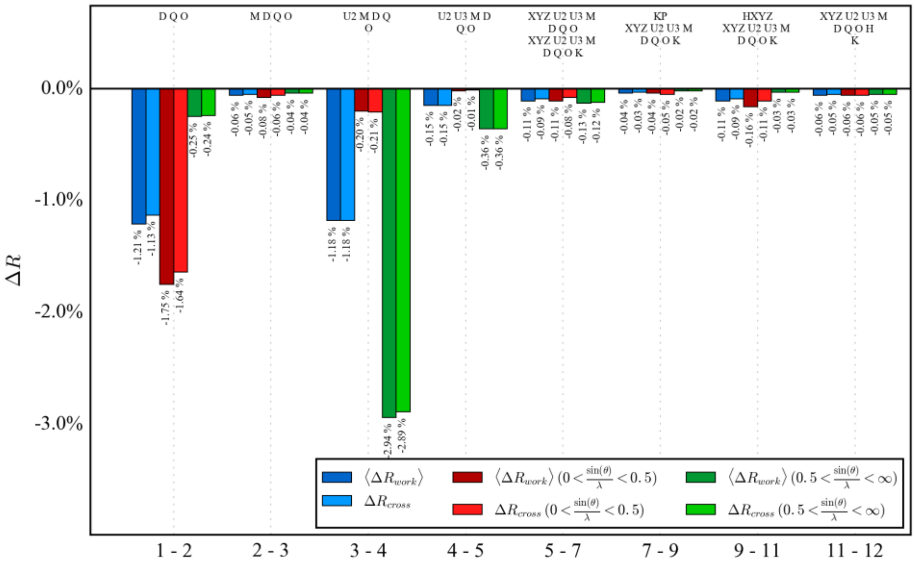

Figure S13. **Cross-validation for steps 1-12.**

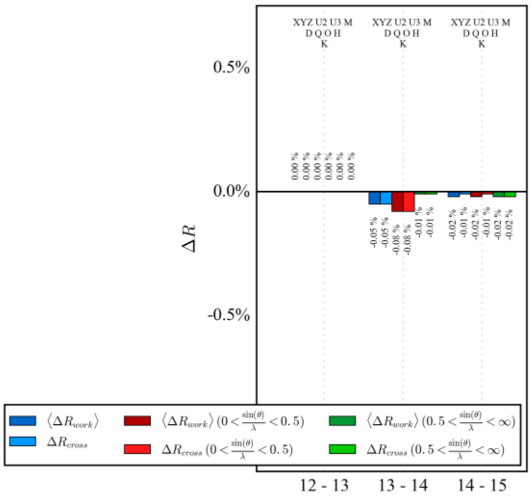

Figure S14. **Cross-Validation for steps 12-15.**

S2.5.2. Data quality[13]–[16]

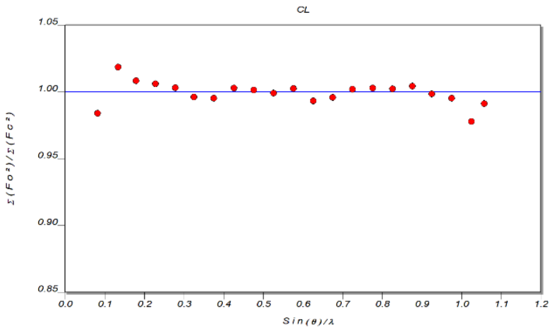

Figure S15. **DRK plot.**

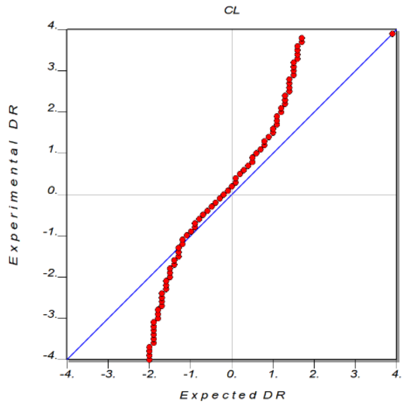

Figure S16. **Normal probability plot.**

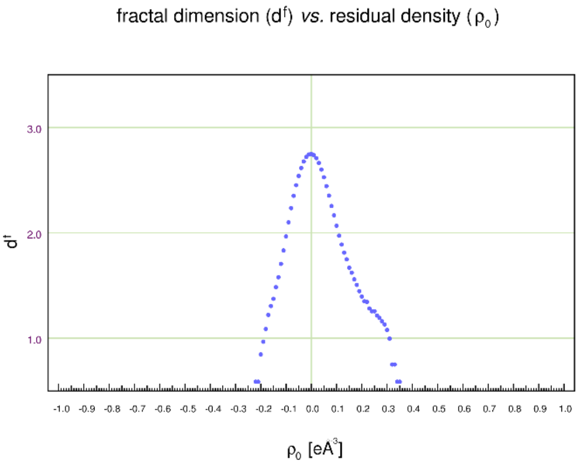

Figure S17. **Henn-Meindl plot.**

S2.5.3. Refinement of anharmonic motion[18],[19]

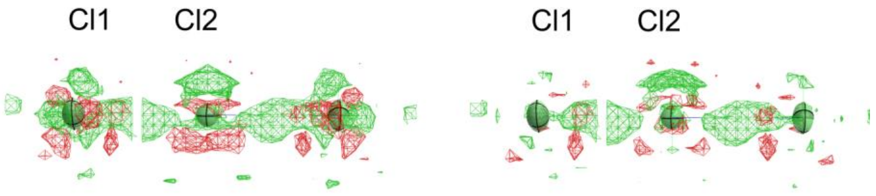

Figure S18. **Residual density before and after anharmonic refinement. The isosurface value is ±0.10 eÅ^{-3}. Positive contours are plotted with green lines and negative contours are plotted with red lines. The graphics were created with MoleCoolQT[19].**

Table S20. The table below shows the minimum data resolution required for meaningful refinement of anharmonic thermal parameters (Gram-Charlier coefficients), for each anisotropic atom.[20]

Atom	Principal M.D.A's (A)			Min. resolution Qn sin(theta)/lambda	
				n =3	n = 4
Cl(1)	0.199	0.164	0.125	0.81	0.94
Cl(2)	0.137	0.133	0.113	1.02	1.17

Table S21. Calculated vibrational probability density function.

Atom	Total integrated negative probability [%]	Total integrated positive probability[%]	Maximum PDF value	Minimum PDF value	Integrated volume for negative probability [$Å^3$]	Integrated volume for positive probability [$Å^3$]
Cl1	0.000	99.999	12451.91	-0.15	0.520	3.820
Cl2	-0.001	100.001	24550.12	-0.44	1.128	3.212

Table S22. Hirshfeld-test[21] after final refinement step. Differences of the mean-square displacement amplitudes (DMSDA) (1 × 10-4 Å2) along atomic vectors.

ATOM	ATOM	DIST	DMSDA
Cl(1)	Cl(2)	2.2826	22
N(1)	C(1)	1.5180	1
N(1)	C(4)	1.5173	2
C(1)	C(2)	1.5159	5
C(2)	C(3)	1.5273	1
C(4)	C(5)	1.5187	6
C(5)	C(4)	1.5261	1

In the case of strongly polar/ionic bonds (Cl_3^-), the rigid-bonds model is not fulfilled.

S2.5.4. Properties

Table S23. Properties at bond critical points evaluated from experimentally determined and quantum-chemically calculated electron densities for 1-3 as well isolated Cl_2 and $[Cl_3]^-$. $R_{(A-B)}$: bond path; $R_{(M-BCP)}$: distance between bond center and BCP (for negative values the BCP is closer to atom A; for positive closer to B); $\rho(r_{BCP})$: electron density at BCP; $\nabla^2\rho(r_{BCP})$: Laplacian at BCP; $G(r_{BCP})$: kinetic energy density at BCP; $V(r_{BCP})$: potential energy density at BCP; $H(r_{BCP})$: total energy density at BCP; ELF_{BCP} electron localisation function at BCP. For other bond lengths see the Supporting Information. Theoretical values obtained by density functional theory (DFT) for trichlorine monoanions embedded in periodic crystals at B3LYP/def2-TZVP level without f-functions are given in square brackets.

Compound	A	B	$R_{(A-B)}$ [Å]	$R_{(M-BCP)}$ [Å]	$\rho(r_{BCP})$ [eÅ^{-3}]	$\nabla^2\rho(r_{BCP})$ [eÅ^{-5}]	$\lvert V\rvert(r_{BCP})/G(r_{BCP})$	$H(r_{BCP})/\rho(r_{BCP})$ [E_h/e]	ELF_{BCP}
**1** $[NMe_3EtCl][Cl_3]$	Cl1	Cl2	2.1075	+0.0075	0.76 [0.82]	5.5 [1.9]	1.51 [1.69]	-0.50 [-0.35]	[0.66]
	Cl2	Cl3	2.5816	-0.0451	0.27 [0.31]	3.0 [2.4]	1.13 [1.13]	-0.07 [-0.08]	[0.25]
**2** $[NMe_4][Cl_3]$	Cl1	Cl2	2.1277	+0.0348	0.84 [0.78]	5.0 [2.1]	1.58 [1.64]	-0.58 [-0.34]	[0.63]
	Cl2	Cl3	2.4989	-0.0512	0.35 [0.36]	3.6 [2.6]	1.20 [1.19]	-0.14 [-0.12]	[0.29]
**3** $[N(nPr)_4][Cl_3]$	Cl1	Cl2	2.2821	+0.0573	0.60 [0.58]	5.0 [2.7]	1.40 [1.41]	-0.38 [-0.23]	[0.48]
$[Cl_3]^-$	Cl1	Cl2	2.3180	-0.0385	[0.52]	[2.7]	[1.37]	[-0.21]	[0.46]
Cl_2	Cl1	Cl1’	1.9900	±0.0000	[1.02]	[0.3]	[1.95]	[-0.44]	[0.76]

Table S24. C–H···Halogen distances (in Å) shorter than the sum of the van der Waals radii[22],[23] in the crystal structures of 1-3. The distances marked with a star are created by a mirror, and the distance marked with a prime are created by a 2-fold axis.

Atom	$[NMe_3EtCl][Cl_3]$ (**1**)	$[NMe_4][Cl_3]$ (**2**)	$[NnPr_4][Cl_3]$ (**3**)
Cl1			**H1A** 2.67559(19)
			**H1B** 2.81519(14)
Cl2	**H4** 2.82237(5)	**H1X** 2.87724(10)	**H4B** 2.72651(3)
	**H4*** 2.82237(5)	**H1X*** 2.87724(10)	**H4B’** 2.72651(3)
Cl3/Cl1‘	**H2X** 2.62934(9)	**H1X** 2.76330(10)	**H1A** 2.67559(19)
	**H2X*** 2.62934(9)	**H1X*** 2.76330(10)	**H1B** 2.81519(14)
	**H2Y** 2.75502(10)	**H1Y** 2.77802(14)	
	**H2Y*** 2.75502(10)	**H1Y*** 2.77802(14)	
	**H3** 2.80456(5)	**H1Z** 2.75791(10)	
	**H3*** 2.80456(5)	**H1Z*** 2.75791(10)	
		**H2Y** 2.74925(15)	
		**H3X** 2.82463(12)	

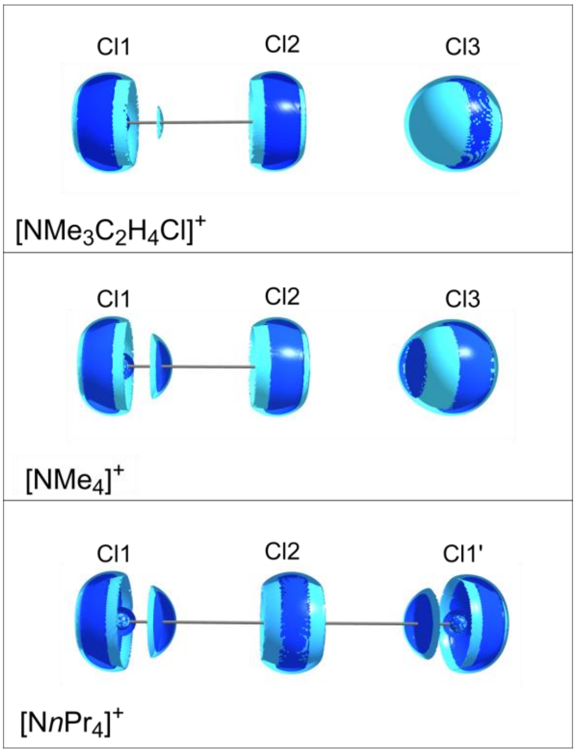

Figure S19. Laplacian of the electron density for 1-3, with the isosurfaces at 14 (light blue) and at 17 e Å^{-5} (dark blue). The charge concentrations form three-dimensional tori around the atoms (indicated in dark blue).

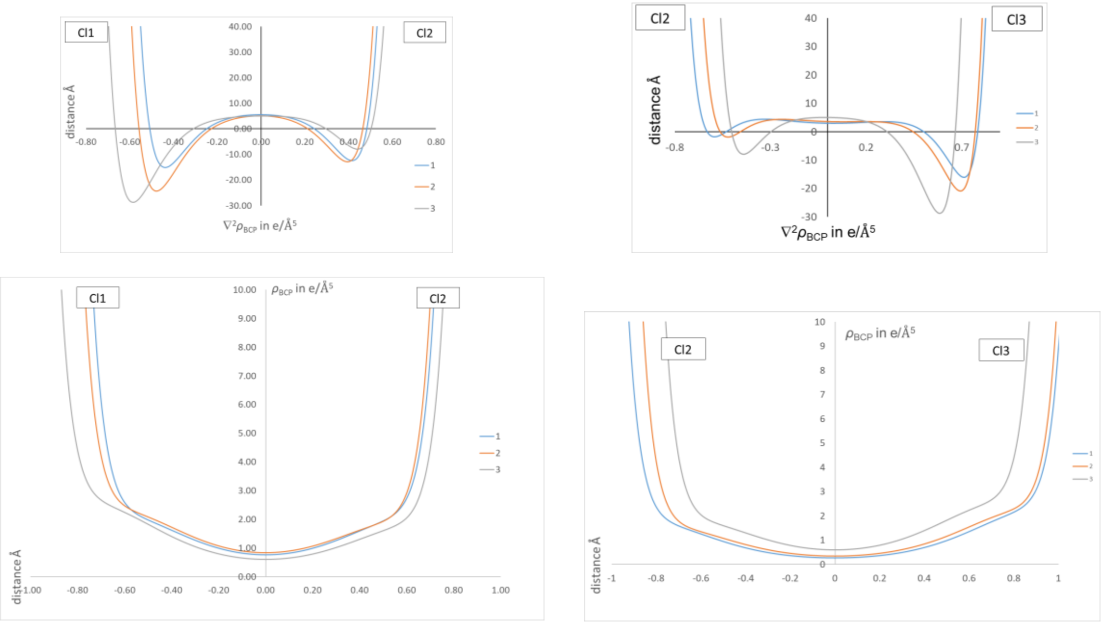

Figure S20. Top: Course of the Laplacian along the BP. Bottom: Course of the electron density along the BP.

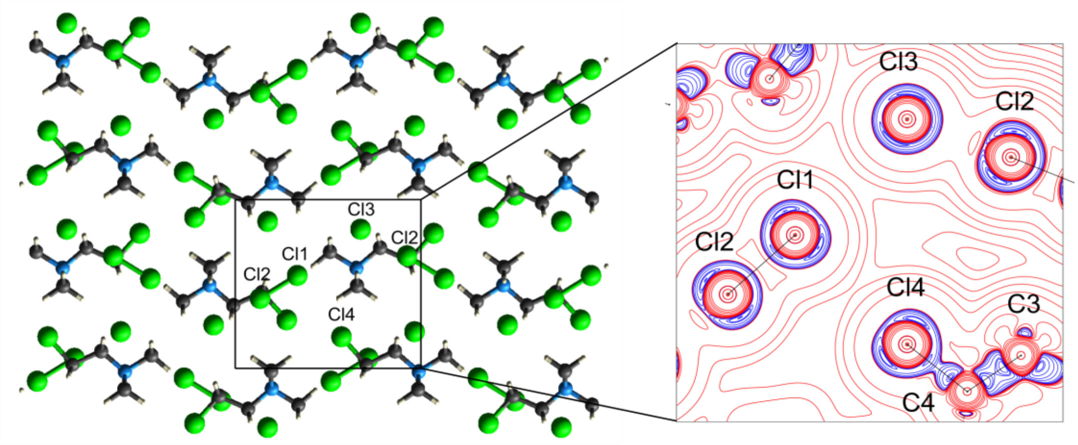

Figure S21. Section of the crystal structure of 1 showing short Cl···Cl contacts in the *bc* plane. The enlarged section (right) shows the Laplacian of the electron density in the same plane with positive values in red, negative values in blue and contours at ±{0.001 0.002 0.004 0.008 0.02 0.04 0.08 0.2 0.4 0.8 2.0 4.0 8.0 14.0 16.0} e Å^{-5}. The graphics were created with CrystalExplorer[24] (right) and XDGRAPH[25] (left), respectively.

1 shows appreciable Cl···Cl intermolecular contacts for which a bond path with a BCP has been located. Figure S21 shows the section of the crystal structure of **1** in projection to *a* and the Laplacian with all Cl atoms in the plane. Adjacent $[Cl_3]^-$ ions share the same layer in the crystal structure and form a zig-zag chain. The Cl3···Cl1 path of 3.7939(2) Å shown in the cut-out in Figure S21 (right) has an electron density $\rho(r_{BCP})$ of 0.03 eÅ^{-3} and a Laplacian $\nabla^2\rho(r_{BCP})$ of 0.3 eÅ^{-5}. The contact of Cl1 to the chlorine atom Cl4 in the $[NMe_3EtCl]^+$ cation has an electron density $\rho(r_{BCP})$ of 0.03 eÅ^{-3} and $\nabla^2\rho(r_{BCP})$ = 0.4 eÅ^{-5}.

S2.5.5. QTAIM parameters for 1

Table S25. Calculated BCPs and local energy density properties for 1. *R*: bond path; $\rho(r_{BCP})$ electron density at BCP; $\nabla^2\rho(r_{BCP})$: Laplacian at BCP; *ε* ellipticity; $G(r_{BCP})$: kinetic energy density at BCP; $V(r_{BCP})$: potential energy density at BCP; $H(r_{BCP})$: total energy density at BCP. The Cl-H bond path were searched in the range of 2.84, which corresponds the sum of the van der Waas radii[22],[23].

A	B	R(A-B) (Å)	R(A-BCP) (Å)	R(B-BCP) (Å)	$\rho(r_{BCP})$ (eÅ^{-3})	$\nabla^2\rho(r_{BCP})$ (eÅ^{-5})	ε	$G(r_{bcp})$ (E_h Å^{-3})	$V(r_{BCP})$ (E_h Å^{-3})	$H(r_{BCP})$ (E_h Å^{-3})
C(1)	H(1X)	1.0771	0.7277	0.3494	1.82	-22.2	0.1	1.14	-3.83	-2.69
C(1)	H(1Y)	1.0771	0.7278	0.3493	1.82	-22.2	0.1	1.14	-3.83	-2.69
C(1)	N(1)	1.5015	0.6135	0.8880	1.59	-14.2	0.0	1.07	-3.14	-2.07
C(2)	H(2X)	1.0772	0.7306	0.3466	1.83	-22.2	0.1	1.16	-3.88	-2.71
C(2)	H(2Y)	1.0771	0.7306	0.3465	1.83	-22.2	0.1	1.16	-3.88	-2.72
C(2)	H(2Z)	1.0770	0.7305	0.3465	1.83	-22.2	0.1	1.16	-3.88	-2.72
C(2)	N(1)	1.5012	0.6069	0.8943	1.63	-14.7	0.0	1.14	-3.31	-2.17
C(3)	H(3)	1.0925	0.7375	0.3550	1.78	-21.0	0.1	1.12	-3.70	-2.59
C(3)	C(4)	1.5185	0.7346	0.7839	1.69	-16.7	0.0	1.15	-3.47	-2.32
C(3)	N(1)	1.5116	0.6236	0.8880	1.59	-12.1	0.0	1.18	-3.21	-2.03
C(4)	Cl(4)	1.7940	0.7873	1.0067	1.15	-3.1	0.1	0.87	-1.95	-1.08
C(4)	H(4)	1.0921	0.7368	0.3553	1.77	-20.8	0.0	1.12	-3.68	-2.57
Cl(1)	Cl(2)	2.1075	1.0613	1.0462	0.76	5.5	0.0	0.77	-1.16	-0.38
Cl(2)	Cl(3)	2.5816	1.2457	1.3359	0.27	3.0	0.0	0.23	-0.26	-0.02
Cl(1)	X4_Cl(3)	3.7938	1.8814	1.9124	0.02	0.3	0.0	0.02	-0.01	0.01
Cl(1)	X1_Cl(4)	3.6822	1.8724	1.8098	0.03	0.4	0.0	0.02	-0.01	0.01
Cl(2)	X3_H(4)	2.8224	1.7979	1.0245	0.04	0.5	0.4	0.03	-0.02	0.01
Cl(3)	X3_H(2X)	2.6303	1.7046	0.9257	0.06	0.6	0.1	0.04	-0.03	0.01
Cl(3)	X3_H(3)	2.8055	1.7949	1.0106	0.04	0.5	0.0	0.03	-0.02	0.01
Cl(3)	X6_H(2Y)	2.7556	1.7690	0.9866	0.04	0.5	0.0	0.03	-0.02	0.01

S2.5.6. QTAIM parameters for 2

Table S26. Calculated BCPs and local energy density properties for 2. *R*: bond path; *ρ*(r_{BCP}) ED at BCP; $\nabla^2\rho(r_{BCP})$: Laplacian at BCP; *ε* ellipticity; *G*(r_{BCP}): kinetic energy density at BCP; *V*(r_{BCP}): potential energy density at BCP; *H*(r_{BCP}): total energy density at BCP. The Cl-H bond path were searched in the range of 2.84, which corresponds the sum of the van der Waas radii[22],[23].

A	B	R(A-B) (Å)	R(A-BCP) (Å)	R(B-BCP) (Å)	ρ(r_{BCP}) (eÅ^{-3})	∇^2ρ(r_{BCP}) (eÅ^{-5})	ε	G(r_{bcp}) (E_h Å^{-3})	V(r_{BCP}) (E_h Å^{-3})	H(r_{BCP}) (E_h Å^{-3})
C(1)	H(1X)	1.0770	0.6799	0.3971	1.86	-21.6	0.1	1.25	-4.01	-2.76
C(1)	H(1Y)	1.0770	0.6801	0.3969	1.86	-21.6	0.1	1.25	-4.01	-2.76
C(1)	H(1Z)	1.0770	0.6799	0.3971	1.86	-21.6	0.1	1.25	-4.01	-2.76
C(1)	N(1)	1.4994	0.6404	0.8590	1.68	-12.8	0.0	1.30	-3.50	-2.20
C(2)	H(2X)	1.0771	0.6796	0.3975	1.86	-22.0	0.1	1.23	-4.01	-2.78
C(2)	H(2Y)	1.0770	0.6849	0.3921	1.83	-21.3	0.1	1.21	-3.90	-2.69
C(2)	N(1)	1.4983	0.6339	0.8644	1.67	-14.3	0.0	1.23	-3.45	-2.23
C(3)	H(3X)	1.0770	0.6850	0.3920	1.83	-21.3	0.1	1.21	-3.90	-2.70
C(3)	H(3Y)	1.0770	0.6796	0.3974	1.86	-22.0	0.1	1.23	-4.01	-2.78
C(3)	N(1)	1.4993	0.6325	0.8668	1.67	-14.2	0.0	1.23	-3.46	-2.23
CL(1)	CL(2)	2.1277	1.0986	1.0291	0.84	5.0	0.0	0.84	-1.33	-0.49
CL(2)	CL(3)	2.4989	1.1817	1.3172	0.35	3.6	0.0	0.30	-0.36	-0.05
CL(3)	X2_H(3X)	2.8274	1.8101	1.0173	0.04	0.5	0.1	0.03	-0.02	0.01
CL(3)	X3_H(2Y)	2.7511	1.7670	0.9841	0.05	0.5	0.1	0.03	-0.02	0.01
CL(3)	X3_H(1Z)	2.7594	1.7919	0.9675	0.05	0.5	0.1	0.03	-0.02	0.01
CL(3)	X8_H(1Y)	2.7803	1.8008	0.9795	0.04	0.5	0.1	0.03	-0.02	0.01
CL(3)	X4_H(1X)	2.7711	1.8002	0.9709	0.05	0.5	0.4	0.03	-0.02	0.01

S2.5.7. QTAIM parameters for 3

Table 27. Calculated BCPs and local energy density properties for 3. *R*: bond path; *ρ*(r_{BCP}) ED at BCP; $\nabla^2\rho(r_{BCP})$: Laplacian at BCP; *ε* ellipticity; *G*(r_{BCP}): kinetic energy density at BCP; *V*(r_{BCP}): potential energy density at BCP; *H*(r_{BCP}): total energy density at BCP. The Cl-H bond path were searched in the range of 2.84, which corresponds the sum of the van der Waas radii[22],[23].

7	B	R(A-B) (Å)	R(A-BCP) (Å)	R(B-BCP) (Å)	ρ(r_{BCP}) (eÅ^{-3})	∇^2ρ(r_{BCP}) (eÅ^{-5})	ε	G(r_{bcp}) (E_h Å^{-3})	V(r_{BCP}) (E_h Å^{-3})	H(r_{BCP}) (E_h Å^{-3})
C(1)	N(1)	1.5185	0.6594	0.8591	1.60	-12.0	0.0	1.21	-3.25	-2.04
C(1)	H(1A)	1.0918	0.7394	0.3524	1.80	-20.9	0.1	1.17	-3.81	-2.64
C(1)	H(1B)	1.0923	0.7399	0.3524	1.80	-20.9	0.1	1.17	-3.81	-2.64
C(1)	C(2)	1.5159	0.8160	0.6999	1.69	-14.8	0.0	1.23	-3.50	-2.27
C(2)	C(3)	1.5279	0.7524	0.7755	1.67	-15.0	0.0	1.19	-3.43	-2.24
C(2)	H(2A)	1.0918	0.7324	0.3594	1.72	-17.8	0.1	1.15	-3.55	-2.40
C(2)	H(2B)	1.0924	0.7329	0.3595	1.72	-17.7	0.1	1.15	-3.54	-2.39
C(3)	H(3A)	1.0773	0.6861	0.3912	1.82	-20.3	0.1	1.23	-3.89	-2.66
C(3)	H(3B)	1.0769	0.6864	0.3905	1.82	-20.2	0.1	1.24	-3.89	-2.65
C(3)	H(3C)	1.0769	0.6865	0.3904	1.82	-20.2	0.1	1.24	-3.89	-2.65
C(4)	C(5)	1.5193	0.8172	0.7021	1.68	-14.6	0.0	1.23	-3.48	-2.25
C(4)	H(4A)	1.0917	0.7394	0.3523	1.81	-21.0	0.1	1.17	-3.82	-2.64
C(4)	H(4B)	1.0926	0.7400	0.3526	1.80	-20.9	0.1	1.17	-3.81	-2.63
C(4)	N(1)	1.5170	0.6514	0.8656	1.62	-12.7	0.0	1.21	-3.31	-2.10
C(5)	C(6)	1.5258	0.7510	0.7748	1.68	-15.1	0.0	1.19	-3.45	-2.25
C(5)	H(5A)	1.0918	0.7324	0.3594	1.72	-17.8	0.1	1.15	-3.55	-2.40
C(5)	H(5B)	1.0928	0.7331	0.3597	1.71	-17.7	0.1	1.15	-3.53	-2.38
C(6)	H(6A)	1.0776	0.6866	0.3910	1.82	-20.3	0.1	1.23	-3.89	-2.65
C(6)	H(6B)	1.0766	0.6856	0.3910	1.82	-20.4	0.1	1.23	-3.89	-2.66
C(6)	H(6C)	1.0772	0.6864	0.3908	1.82	-20.2	0.1	1.24	-3.89	-2.65
CL(1)	CL(2)	2.2821	1.1983	1.0838	0.60	5.0	0.0	0.58	-0.81	-0.23
CL(1)	H(1A)	2.6773	1.6476	1.0297	0.07	0.8	0.1	0.04	-0.04	0.01
CL(1)	X3_H(1B)	2.8175	1.8013	1.0162	0.04	0.5	0.2	0.03	-0.02	0.01
CL(2)	X3_H(4B)	2.7277	1.7435	0.9842	0.05	0.6	0.1	0.04	-0.03	0.01

S3. Laplacian and ELF for isolated Cl_2 and $[Cl_3]^-$

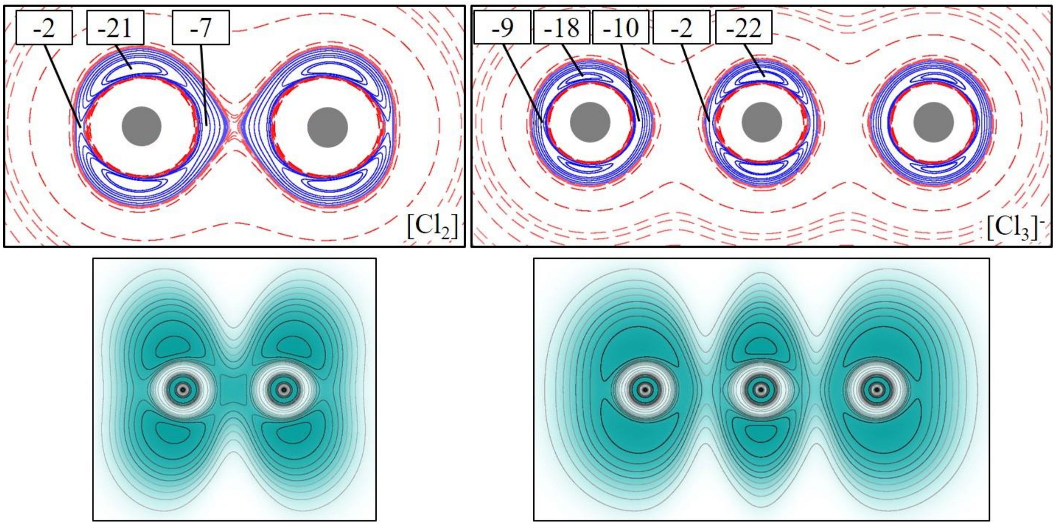

Figure S22. Top: Calculated Laplacian of the electron density for isolated Cl_2 (left) and $[Cl_3]^-$ (right). Positive values are in red, negative values in blue and contours are ±{0.001 0.002 0.004 0.008 0.02 0.04 0.08 0.2 0.4 0.8 2.0 4.0 8.0 14.0 16.0} e Å^{-5}. The values of minima in the Laplacian are indicated. Bottom: Calculated ELF for isolated Cl_2 (left) and $[Cl_3]^-$ (right). Values range from 0.0 (white) to 1.0 (dark cyan) with contours plotted from 0.1 to 1.0 in intervals of 0.1. The graphics was created with Gri[19].

S4. Computational Details

All periodic solid-state calculations for systems **1**-**3** as well as single-point molecular calculations for isolated Cl_2 and $[Cl_3]^-$ (with different bond distances) were performed with the CRYSTAL17[26] program, using the B3LYP DFT functional and employing the Gaussian-type atomic basis set def2-TZVP[27]. The first Brillouin zone was sampled using an 8×8×8 Monkhorst-Pack grid. To facilitate convergence, the Coulomb and exchange integral thresholds were sufficiently tightened with the TOLINTEG keyword to values of 12, 10, 10, 30 and 80. Partial structure optimizations at CCSD(T) level with the def2-TZVP basis set for isolated $[Cl_3]^-$, as used in the discussion of the properties at bond critical points (Figure 2), were performed with the MOLPRO2019[28] program. In these calculations, one Cl-Cl bond distance was fixed while the other was optimized. QTAIM Analysis was performed with the TOPOND code developed by Gatti[29] and recently implemented in CRYSTAL17; data for 2D-maps of the Laplacian and ELF were calculated with the CRYSTAL17 program and visualized with Gri[30]. Both QTAIM analysis and the calculation of the Laplacian and ELF required the removal of all f-functions from the basis set.

S5. References

[1] T. Schulz, K. Meindl, D. Leusser, D. Stern, J. Graf, C. Michaelsen, M. Ruf, G. M. Sheldrick, D. Stalke, *J. Appl. Cryst.* **2009**, *42*, 885.
[2] T. Kottke, D. Stalke, *J. Appl. Cryst.* **1993**, *26*, 615.
[3] a) Bruker AXS Inc., *SAINT*, Madison, **2016**; b) G. M. Sheldrick, *SADABS 2014/4*, Göttingen, **2014**.
[4] Bruker AXS Inc., *APEX3*. *Crystallographic Software Suite*, Madison, WI, USA, **2016**.
[5] L. Krause, R. Herbst-Irmer, G. M. Sheldrick, D. Stalke, *J. Appl. Cryst.* **2015**, *48*, 3.
[6] G. M. Sheldrick, *Acta Crystallogr.* **2015**, *A71*, 3.
[7] G. M. Sheldrick, *Acta Crystallogr.* **2015**, *C71*, 3.

[8] C. B. Hübschle, G. M. Sheldrick, B. Dittrich, *J. Appl. Cryst.* **2011**, *44*, 1281.
[9] N. K. Hansen, P. Coppens, *Acta Crystallogr. Sec. A* **1978**, *34*, 909.
[10] A. Volkov, P. Macchi, L. J. Farrugia, C. Gatti, P. R. Mallinson, T. Richter, T. Koritsanszky, *XD2016. A Computer Program Package for Multipole Refinement, Topological Analysis of Charge Densities and Evaluation of Intermolecular Energies from Experimental and Theoretical Structure Factors*, **2016**.
[11] R. F. W. Bader, *Atoms in Molecules*. *A Quantum Theory*, Clarendon Press, Oxford, New York, **1990**.
[12] L. Krause, B. Niepötter, C. J. Schürmann, D. Stalke, R. Herbst-Irmer, *IUCrJ* **2017**, *4*, 420.
[13] L. Krause, R. Herbst-Irmer, D. Stalke, *J. Appl. Cryst.* **2015**, *48*, 1907.
[14] V. V. Zhurov, E. A. Zhurova, A. A. Pinkerton, *J. Appl. Cryst.* **2008**, *41*, 340.
[15] K. Meindl, J. Henn, *Acta Crystallogr. Sec. A* **2008**, *64*, 404.
[16] Adam Stash, *DRKplot*, Moscow, **2007**.
[17] S. C. Abrahams, E. T. Keve, *Acta Crystallogr. Sec. A* **1971**, *27*, 157.
[18] R. Herbst-Irmer, J. Henn, J. J. Holstein, C. B. Hübschle, B. Dittrich, D. Stern, D. Kratzert, D. Stalke, *J. Phys. Chem. A* **2013**, *117*, 633.
[19] C. B. Hübschle, B. Dittrich, *J. Appl. Cryst.* **2011**, *44*, 238.
[20] W. F. Kuhs, *Acta Crystallogr. Sec. A* **1992**, *48*, 80.
[21] F. Hirshfeld, *Acta Crystallogr. Sec. A* **1976**, *32*, 239.
[22] R. S. Rowland, R. Taylor, *J. Phys. Chem.* **1996**, *100*, 7384.
[23] A. Bondi, *J. Phys. Chem.* **1964**, *68*, 441.
[24] C. F. Mackenzie, P. R. Spackman, D. Jayatilaka, M. A. Spackman, *IUCrJ* **2017**, 575.
[25] A. Volkov, P. Macchi, L. J. Farrugia, C. Gatti, P. R. Mallinson, T. Richter, T. Koritsanszky, *XD2006*. *XD2006*, **2006**.
[26] R. Dovesi, A. Erba, R. Orlando, C. M. Zicovich-Wilson, B. Civalleri, L. Maschio, M. Rérat, S. Casassa, J. Baima, S. Salustro et al., *Wiley Interdiscip. Rev. Comput. Mol. Sci.* **2018**, *8*, e1360.
[27] F. Weigend, R. Ahlrichs, *Phys. Chem. Chem. Phys.* **2005**, *7*, 3297.
[28] a) H.-J. Werner, P. J. Knowles, G. Knizia, F. R. Manby, M. Schütz, *Wiley Interdiscip. Rev. Comput. Mol. Sci.* **2012**, *2*, 242; b) H.-J. Werner, P. J. Knowles, G. Knizia, F. R. Manby, M. Schütz, P. Celani, T. Korona, R. Lindh, A. Mitrushenkov, G. Rauhut et al., *MOLPRO, a package of ab initio programs, version 2019.1*, http://www.molpro.net., **2012**.
[29] C. Gatti, *TOPOND-96: An electron density topological program for systems in N (N=0-3) dimensions*. *User's manual, CNR-CSRSRC*, Milano, Italy, **1996**.
[30] D. E. Kelley, *gri - scientific graphic program, version 2.12.26*, **2017**.
